# Health- Related Quality of Life for Multiple Myeloma Patients with Bone Metastases in Indonesia: A Cross-Sectional Study

**DOI:** 10.31557/APJCP.2019.20.10.3161

**Published:** 2019

**Authors:** Nutrisia Aquariushinta Sayuti, Tri Murti Andayani, Dwi Endarti, Kartika Widayati Taroeno-Hariadi

**Affiliations:** 1 *Doctoral Program, Faculty of Pharmacy,*; 3 *Division of Hematology and Medical Oncology, Department of Internal Medicine, Faculty of Medicine, Public Health and Nursing, Universitas Gadjah Mada, Yogyakarta,*; 2 *Indonesian Herbal Medicine Department, Health Polytechnic of Surakarta, Ministry of Health Indonesia, Surakarta, Indonesia.*

**Keywords:** HRQoL, cross-sectional, multiple myeloma, bone metastases, Indonesia

## Abstract

**Objective:**

Multiple myeloma (MM) with bone metastases causes a skeletal-related event (SRE), and decreases health-related quality of life (HRQoL). HRQoL needs to be evaluated for health technology assessment (HTA). Furthermore, HRQoL is calculated as a health state utility and is used in the Markov Model for HTA. Therefore, this study aimed to describe the HRQoL of MM patients with bone metastases, using The EuroQol five-dimension five levels (EQ -5D-5L) in Indonesia.

**Methods:**

a cross-sectional, multicenter study for MM patients with bone metastases (aged over 18 years old) that consulted a physician between November 2018 - May 2019 was conducted. The calculated HRQoL illustrated the health state utility, which was assessed using the EQ-5D-5L questionnaire, with the Indonesian value set. In addition, Mann-Whitney analyses were performed to determine the difference in utility scores at different points within the Durie-Salmon staging system and skeletal condition.

**Results:**

in 93 patients who completed the questioner, pain was their major concern with prevalence of over 60% (all levels inclusive). Moreover, the mean utility of patients in stage II and III were 0.735 (SD = 0.205) and 0.383 (SD = 0.555), and those without SRE was 0.753 (SD = 0.213) while patients with SRE was 0.302 (SD = 0.562). Therefore, the lessened values were observed at stage III and SRE condition (p<0.05).

**Conclusion:**

MM patients with bone metastases have poor HRQoL, with pain as the most frequently reported challenge, which is associated with an advanced stage of MM and SRE event.

## Introduction

Multiple myeloma (MM) is the malignant neoplasms of plasma cells that accumulate in the bone marrow, causing damage, and the continuous creation of millions of non-required new “myeloma” cells. These produced cells are not able to make immunoglobulins, but they produce monoclonal proteins, or excessive amounts of M protein (Multiple Myeloma Indonesia, 2016). In addition, the disease is characterized based on the clinical signs such as monoclonal components by serum or urine protein electrophoresis, over 10% clonal plasma cells on bone marrow examination or a biopsy-proven plasmacytoma, abnormal bones on radiograph results, and evidence of end-organ damage termed CRAB criteria (hypercalcaemia, renal insufficiency, anaemia or bone lesions) (Dispenzieri et al., 2004; Moreau et al., 2013).

The prevalence of MM was reported to be 1.8% of cancers and more than 17% of hematologic malignant incidences in United State America (USA). Geriatrics over 65-74 years of age were often diagnosed with it (median age of 69 years old). The American Cancer Society estimated about 30,280 new cases with 12,590 deaths in USA in 2017 (NCCN, 2017), while in Indonesia, the occurrence of blood cancer was 0.8% and MM ranks second after leukaemia (Dewi, 2017; Multiple Myeloma Indonesia, 2016). Besides, it was common in men compared to women, at a ratio of 1.4: 1. Therefore, the first descendants of these patients have an increased risk of developing it by almost 3.7 times (Suega and Sjah, 2009).

Bone manifestations (osteopenia or osteolytic lesions) can develop in 85% of patients. It possibly causes Skeletal-Related Events (SRE), including fractures, spinal cord compression, significant hypercalcemia and pain, further requiring radiation, chemotherapy or surgical intervention (NCCN, 2017). Furthermore, the psychosocial burden often felt by patients is due to the condition, duration of illness, and low quality of life and health status (Kvam and Waage, 2015). 

The main goal of therapy involves prolonging survival, entirely and also to improve health-related quality of life (HRQoL). The study of HRQoL with EQ-5D-3L by Hatswell et al., (2016) stated that patients with newly diagnosed untreated MM manifested low utility (0.46). The utility was increased to approximately 0.60 while receiving the first two treatment lines, and falling to about 0.55 beyond. In addition, Stem Cell Transplants (SCT) is associated with a 0.13 increase in utility, and also while controlling the number of prior treatment lines (Hatswell et al., 2016).

The evaluation of HRQoL is very dependent on the tools used and the interpretation of results. The instrument is needed in the form of a standardized questionnaire, with the ability to explore patient perspectives regarding the health condition (Kvam and Waage, 2015). Furthermore, HRQoL is needed to develop a health technology assessment (HTA). Cost-utility analysis (CUA) for new or existing medical intervention must be employed to ensure HTA. CUA is used to evaluate health-related quality-of-life (HRQoL) outcomes and to compare costs and outcomes between different medical intervention in terms of cost per quality-adjusted life-years (cost per QLAY). A QALY is obtained by integrating a health state utility function (Purba et al., 2017). The health state utility is applied in economic modelling to represent the value for patients (Hatswell et al., 2016). Therefore, these instruments (questionnaires) must require the setting of a societal based value set. The EuroQol five-dimensions five-levels (EQ-5D-5L) is a generic instrument with the value set for Indonesia.

EQ-5D-5L instrument in Indonesian language version is provided by EuroQol Group (Euroqol, 2017). EQ-5D-5L was chosen because it has been established as a measurement instrument for utility in HTA in Indonesia (Ministry of Health Indonesia, 2017). In 2017, the Indonesian version of EQ-5D-5L was translated, validated, standardized, evaluated and published by Purba et al (2017) for use as an HRQoL tool by all HTA and HRQoL studies in Indonesia (Purba et al., 2017). 

Meanwhile, the study of HRQoL with EQ-5D-5L for MM patient has been done in various country. Leunis et al (2019) stated that the average EQ-5D-5L utility was 0.67 (95% CI: 0.64–0.70). The average EQ-5D-5L utility ranged from 0.64 (95% CI: 0.57–0.71) for British patients to 0.74 (95% CI: 0.59–0.91) for French patients. After correcting for patient and disease characteristics, Dutch and Belgian patients reported a significantly higher EQ-5D-5L utility than German and British patients (Dutch versus German: ß = 0.098, p-value = 0.039, Dutch versus British ß = 0.110, p-value = 0.051, Belgian versus German ß = 0.125, p-value = 0.078, Belgian versus British ß = 0.136, p-value = 0.076). Similar differences between nationalities were found for the global quality of life score. Quality of life in MM patients differs between nationalities across Europe (Leunis et al, 2019) 

This assessment has never been conducted. Therefore, the main objective of this study was to describe HRQoL in MM patients with bone metastases in Indonesia. Furthermore, the study was evaluated utility that was classified by stage II and III Durie-Salmon staging system and skeletal condition. Statistical analysis was performed to test the differences stage and skeletal condition associated with differences in utility.

## Materials and Methods

*Study subjects*

A cross-sectional questionnaire survey was conducted at Dr. Sardjito National Hospital Yogyakarta and Dr. Kariadi National Hospital Semarang, and the data were collected between November 2018 and May 2019.

The study subjects were the entire population of MM patients with bone metastases within the two hospitals that fulfilled the inclusion and exclusion criteria over the period 2016– 2018, invited to enrol for the investigation.

The inclusion criteria of the patients were adults (>18 years old) suffering from MM bone metastases. Patients may have comorbid or without comorbid. Patients have complete medical record data (age, gender, disease stage, number of comorbidities, skeletal conditions, duration of illness, educational level, employment status and marital status). Meanwhile, patients were excluded from instances where they were not receiving specific therapy or where there is an unwillingness to become respondents in this study.

*Data collection and analysis*

Potential participants that consulted a physician between November 2018 - May 2019 were provided with information about the research. The completion of the EQ-5D-5L questionnaire by participants was carried out after their willingness was obtained by signing the informed consent sheet. Therefore, in cases where they had difficulty reading, the officer read the questionnaire and assisted in filling. Some participants also had difficulty in understanding the questionnaire, so that face to face interviews was conducted.

**Table 1:** Basic Characteristic of Patient

Characteristic	Total (N = 93)
Proportion (%)	93 (100.0)
Age (years), mean±SD	58.4 ± 8.9
Age distribution	
30 – 50	18 (19.4)
>50	75 (80.6)
Gender distribution	
Male	62 (66.7)
Female	31 (33.3)
Duration of illness (Q1-Q3)	2 – 64
Durie Salmon Stage	
II	30 (32.3)
III	63 (67.7)
Number of comorbidities*	
0	41 (44.1)
≥1	52 (55.9)
Skeletal Condition	
No. SRE	40 (43.0)
SRE	53 (57.0)
Education level	
Not attending school	2 (2.2)
Elementary school	21 (22.6)
Junior high school	12 (12.9)
Senior high school	34 (36.5)
University degree	24 (25.8)
Employment status	
Unemployment	31 (33.3)
Part-time job	2 (2.2)
Self-employed	22 (23.6)
Paid-employed	38 (40.9)
Marital status	
Married	93 (100.0)
Single	0 (0.0)

Note: Data are presented as n (%) unless otherwise indicated, comorbidities included: history of neuropathy, renal insufficiency, hypertension, hypercholesterolemia, diabetes mellitus, previous deep vein thrombosis, respiratory failure, coronary heart disease, heart failure, other.

**Table 2:** Problems Reported by Respondents in the Five Dimensions of EQ-5D-5L

Problems	Mobility	Self-care	Usual activity	Pain/discomfort	Anxiety/ depression
No	29 (31.2)	61 (65.6)	34 (36.6)	24 (25.8)	45 (48.4)
Slight	33 (35.5)	9 (9.7)	30 (32.3)	36 (38.7)	28 (30.1)
Moderate	16 (17.2)	8 (8.6)	10 (10.8)	20 (21.5)	13 (14.0)
Severe	3 (3.2)	2 (2.2)	7 (7.5)	7 (7.5)	6 (6.5)
Extreme	12 (12.9)	13 (14.0)	12 (12.9)	6 (6.5)	1 (1.1)

**Table 3:** EQ-5D-5L Index Score Classified by Durie-Salmon Stage II, III and Skeletal Condition

Characteristics	Mean	SD	95% CI of mean		SE	p-values
			Lower	Upper		
Durie-Salmon Stage						0.001
II	0.735	0.205	0.658	0.811	0.038	
III	0.383	0.555	0.243	0.522	0.070	
Skeletal condition						0.000
No. SRE	0.753	0.213	0.685	0.821	0.034	
SRE	0.302	0.562	0.148	0.457	0.077	
Total	0.496	0.498	0.394	0.599	0.052	

**Figure 1:**
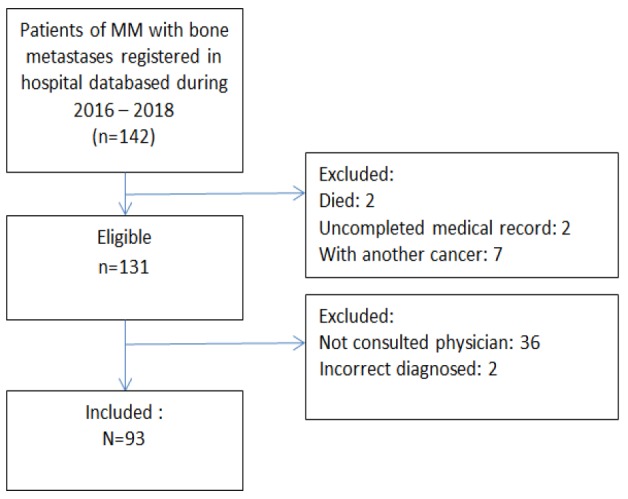
Flow Chart of MM Patients with Bone Metastases Studied

Clinical and socio-demographic characteristics of patients were obtained from the patient’s medical record, using a case report form (CRF). The CRF reported their age, gender, disease stage, number of comorbidities, skeletal conditions, duration of illness, educational level, employment status and marital status. Stage of MM can be assessed by two types of staging system, which are Durie-Salmon staging system and International Myeloma Working Group Staging (IMWG) System (NCCN, 2017). However, the Durie-Salmon staging system was used because it is commonly used in Indonesia.

The EQ-5D-5L that was used for evaluation is Indonesian language version of EQ-5D-5L that validated by Purba et al., (2017). The questionnaire classified health status of subjects into five dimensions, including mobility, self-care, usual activities, pain/discomfort and anxiety/ depression. Furthermore, each was rated on a five-level scale, encompassing: no problems, slights problems, moderate problem, severe problem and extreme problem. Hence, the combination of HRQoL state with 11111 indicated “no problem at all” while 55555 indicated “extreme problems” in all five dimensions. Each combination provided a utility score or value set. Besides, Purba et al., (2017) also stated that the maximum value was 1.000 for full health (health state ‘11111’), followed by the health state ‘11112’, with 0.921, and the minimum was -0.865, for the worst state (‘55555’) (Purba et al., 2017).

Data on patient characteristics were presented in the frequency distribution, except data on age, which was presented in mean (SD), and that of illness duration, displayed in a range. The top 3 ranks of the health status of EQ-5D-5L that felt by the patient were described. The best health state and the worst health state were also reported. The problems in the five dimensions of EQ-5D-5L were sorted from the most to the least informed. 

For statistical model, the dependent variable was the EQ-5D-5L utility score, while the independent variable was the Durie-Salmon stage and the skeletal conditions (no SRE or SRE). Moreover, the EQ-5D-5L utility score followed a non-normal distribution (Kolmogorov-Smirnov test p <0.05). The Mann Whitney test was applied to determine their differences with varying characteristics of Durie-Salmon stage and skeletal conditions. Hence, all statistical analyses were conducted using SPSS V.16, with a p-value less than 0.05, indicating statistical significance. 

## Results

The medical record showed 142 patients were identified for this study, of whom 131 patients were eligible for inclusions. Only 93 patients that consulted a physician between November 2018 and May 2019 so that finally, 93 patients took part in the study (Figure 1). 

The characteristic of the patient was shown in Table 1. Overall mean age of 58.34 years (range: 37 - 79 years), most respondents were male, age above 50 years, and also at the third Durie-Salmon stage and suffering SRE. Meanwhile, the duration of illness was 2 - 64 months. All patients were married. More than half of the patient had high education level (passed senior high school education), and only 40.9% of patients had a formal occupation.

MM patients in Indonesia reported the top 3 rankings of health states. The first rank was 11111 (6.5%) that indicated no problem in all EQ-5D-5L descriptive system. It was followed by 11222 (5.4%) that denoted no problems in the dimension of mobility and self-care but having moderate problems of usual activities, and 21111 (5.4%) that designated moderate problems in mobility but none in self-care, usual activities, pain/discomfort and anxiety/depression. The third rank was 11112 (4.3%) that showed no problems in mobility, self-care, usual activities, and pain/discomfort, and moderate problems in the dimension of anxiety/depression. Meanwhile, the best health state (11111) was only perceived by respondents in stage II and without SRE, while 55555 (the worst health state) were only perceived by patients with SRE (1.1 %), further indicating extreme problems in all dimension.

Pain/ discomfort was more frequently reported in the total respondents (74.2%), followed by mobility (68.8%), usual activity (63.4%), anxiety/depression (51.6%) and self-care (33.4%). Moreover, six of the 93 patients (6.5%) reported the absence of problems in all dimensions (Table 2). 

The respondents had a mean utility score of 0.496 (SD =0.498) (Table 3). The results indicated reduction of utility score MM patients with bone metastases at stage III and with SRE, as later stages of MM and the presence of SRE possessed lower scores in comparison with the others (p <0.05). 

## Discussion

Various treatment, side effect and health technologies influence the health status of cancer patients and their social and emotional well being (Setiawan et al., 2018). The survival of MM patients is probably over 15 years, and the goal of therapy did not only prolong survival but also increased HRQoL (Kvam and Waage, 2015). Furthermore, health economics increasingly focus on this aspect, in addition to the clinical outcomes of patients (Fitzpatrick and Davies, 1998). However, the existence of this related information is highly limited, and the benefits in real-time clinical practice are unclear (Despiégel et al., 2019).

This study, therefore, identified patient characteristics and provided the novel HRQoL data in MM patients with bone metastasis in Indonesia. The research was only done with 93 participants because of the low MM incident in the two hospitals. MM incident in Indonesia was small. Globocan (2018) stated that the new cases of MM were 2,717 cases a year. The new case number was the 20^th ^rank of cancer incident in Indonesia, with a five-year prevalence of 5,884 (World Health Organization, 2018). This research also showed its prevalence to be more common in men than women, which is in concordance with world data, Globocan (2018) that stated male incidents to be higher than females (Bray et al., 2018). 

Conversely, the investigation also showed that patients were mainly above 50 years old, which is in accordances with the Sutandyo et al., (2015) that stated the incidence to occur at over 60 years of age. Pain/ discomfort was the most frequently reported problems. It agreed with the result of the study by Mohebbifar et al., (2015), which showed pain to be one of the poor quality of life signs and symptoms, while the physical function was the second functional status of the quality of life in cancer patients. These findings were also similar to what was found by the previous study in France. Despiégel et al., (2019) stated that symptom score for fatigue and pain were particularly high in MM patients with supportive care as measured using EORTC QLQ-C30 (Despiégel et al., 2019). EORTC QLQ-C30.

The study showed that variations in HRQoL occurred at different Durie-Salmon stages and skeletal conditions. It was manifest that the progression in disease and the presence of SRE can decrease the patient’s HRQoL, and cause a high risk of fracture. Furthermore, this subsequently leads to an elevation in direct medical costs, and a decline in their quality of life (Fardellone et al., 2010) 

Patients with SRE happened to be more numerous than those without, as some had experienced it as an initial symptom. This manifestation caused them to visit the hospital and obtained a proper diagnosis and initiate therapy. Sultan et al., (2016) also stated that the majority of 61 MM patients presented fatigue (81.9%) and backaches (80.3%). The radiological survey showed 78.6% of them reported bone lesions included punched-out lytic lesions, osteoporosis or fractures.

Furthermore, the results of this study indicated the utility of MM patients with SRE (0.302) to be worse than the outcome of Delea et al., (2012), which stated utility value of MM patients with post-progression survival was 0.485. In addition, the score indicated extreme problems in MM patients with bone metastases in Indonesia. The data were cross-sectional as it did not show utility for each respondents’ disease course. Furthermore, the different values obtained between the initial occurrence of SRE and at a certain period after were not examined in this study. Therefore, patients may experience severe physical and mental burden initially, which may lead to the low utility. In addition, the results of a systematic review by Davis et al., (2016), showed the utility of post-fracture patients at the beginning of 4 weeks (0.18) to be lower than in those after 4 months (0.62), although no significant elevation was observed in 12 months (0.67), which tends to decrease subsequently within the next 24 months (0.59).

Our research only classified HRQoL by Durie-Salmon stage and skeletal condition as to health state since this study was part of a pharmacoeconomic study that needs health state utility data. Indonesian culture and belief may be related with the poor HRQoL. Setyowibowo et al., (2018) studied about HRQoL in Indonesian women with breast cancer syptoms before definitive diagnosis. They analysed the association between the demographis variables and HRQoL. The study stated that higher education and monthly income were associated with HRQoL. The higher levels of education and income were associated with more favorable physical, social, and environmental dimensions of quality of life compared to those with lower levels of education. High educated patients or high socio-economic patients may lead to better access to information and health services, and the result, these patients may have fewer problems and feel less uncertain.

The differences in nationality and spiritual health may cause differences in HRQoL. Leunis et al., (2019) stated that HRQoL in MM patients differs between nationalities across Europe. Unfortunately, the study did not identify the reason for the differences between nationalities. Mohebbifar et al., (2015) stated that spiritual health should be a factor that improves HRQoL patients with cancer, so a combination of care therapies and spiritual interventions is a priority in cancer treatment. 

In conclusion, patients had poor HRQoL, and pain/discomfort was the most frequently reported problems, associated with the more advanced stage of MM and the presence of SRE. In addition to the methods applied in this research, the limitations of the study were the inadequate number of participants, as it was only conducted in two national hospitals with cancer centres in two provinces of Indonesia; hence a generalization of results needs to be with caution. Further study with a cohort and more exclusion criteria regarding specific comorbidity still required. However, based on existing knowledge, this report is the first complete publication of real data on HRQoL in MM bone metastases patients, using Indonesia EQ-5D-5L value set. 
